# The One Health Concept

**DOI:** 10.3389/bjbs.2024.12366

**Published:** 2024-02-15

**Authors:** Sarah J. Pitt, Alan Gunn

**Affiliations:** ^1^ School of Applied Sciences, University of Brighton, Brighton, United Kingdom; ^2^ School of Biological and Environmental Sciences, Liverpool John Moores University, Liverpool, United Kingdom

**Keywords:** One Health, laboratory services, neglected tropical diseases, zoonoses, antimicrobial resistance

## Abstract

The concept of One Health has been developed as the appreciation that human health is intricately connected to those of other animals and the environment that they inhabit. In recent years, the COVID-19 pandemic and noticeable effects of climate change have encouraged national and international cooperation to apply One Health strategies to address key issues of health and welfare. The United Nations (UN) Sustainable Development Goals have established targets for health and wellbeing, clean water and sanitation, climate action, as well as sustainability in marine and terrestrial ecosystems. The One Health Quadripartite comprises the World Health Organization (WHO), the World Organization for Animal Health (WOAH—formerly OIE), the United Nations Food and Agriculture Organization (FAO) and the United Nations Environment Programme (UNEP). There are six areas of focus which are Laboratory services, Control of zoonotic diseases, Neglected tropical diseases, Antimicrobial resistance, Food safety and Environmental health. This article discusses the concept of One Health by considering examples of infectious diseases and environmental issues under each of those six headings. Biomedical Scientists, Clinical Scientists and their colleagues working in diagnostic and research laboratories have a key role to play in applying the One Health approach to key areas of healthcare in the 21st Century.

## Introduction

One Health [1] is an approach to investigating infectious diseases which acknowledges that humans, animals, plants, and the environment are closely interlinked. In the mid-20th century, a veterinary surgeon in the United States called Calvin Schwabe compared approaches to human and animal health and welfare and suggested the concept of “One Medicine” [2]. He highlighted the integrated, cross-disciplinary perspective which members of his profession could contribute to general medicine. He also advocated involvement of social sciences and enhancing communication skills to improve working together with the community in efforts to control infectious diseases [2].

During the 21st Century this idea was developed further, to encompass the health of the wider ecosystem, including that of plants, wild animals, and geographical area. The Manhattan Principles of Conservation, published in 2004, called for a more integrated view of interactions between humans and animals [3]. The 2002–2004 global outbreak of Severe Acute Respiratory Syndrome coronavirus (SARS-CoV-1) sharply highlighted the risks posed to humans by zoonoses [4]. The term “One Health” was first coined at around this time. This concept acknowledges that the health of humans, animals, their behaviour, and their environment are all closely interlinked [5]. To paraphrase the first century poet, Juvenal (“You should pray for a healthy mind in a healthy body”) [6], a healthy human needs a healthy ecosystem. In this analogy the ecosystem translates as everything from the microorganisms living in our gut to the home we live in, the way we live our lives, our immediate environment, the wider environment, and the world as a whole [7]. To give an indication of how interest in this approach is developing, a scientific journal “Science in One Health” [8] was established in 2022 that is devoted to the topic. Although the term One Health has only been in general use since 2004, many of the linkages it explores have a long pedigree with examples to be found in both Western and Asian treatises dating back hundreds and even thousands of years [1, 4, 7].

The United Nations (UN) Sustainable Development Goals [9] are relevant to One Health as they include targets for health and wellbeing, clean water and sanitation, climate action, as well as sustainability in marine and terrestrial ecosystems. Recognizing the importance of cross disciplinary, multi-national working, four global organizations have now agreed to form the One Health Quadripartite: The World Health Organization (WHO), the World Organization for Animal Health (WOAH—formerly OIE), the UN Food and Agriculture Organization (FAO) and the UN Environment Programme (UNEP). They have identified six areas to focus on [10], namely:

• Laboratory services
• Control of zoonotic diseases
• Neglected tropical diseases
• Antimicrobial resistance
• Food safety
• Environmental health

Microbiologists working in both diagnostic and research laboratories contribute to all these areas. This review will consider each of these in turn although, as outlined above, they are all interconnected to varying degrees.

### One Health Approach to Laboratory Services

Laboratory investigations are a key element for the diagnoses for infectious diseases [11]. This is because their symptoms can often be non-specific (e.g., diarrhoea, pyrexia, jaundice) and full identification of the causal organism is important to determine treatment options. Many human pathogens are either acquired zoonotically or they have close relatives which affect other animals. Thus, the One Health approach encourages medical and veterinary laboratory scientists to cooperate in the development of laboratory testing protocols. For example, many protozoan parasites of *Leishmania* spp. affect humans, as well as domestic and wild animals [12]. They are transmitted via sandflies of the *Phlebotomus* or *Lutzomyia* genera. There are at least 53 species of *Leishmania*, of which 20 infect humans. The WHO estimates that 1 billion people live in areas endemic for *Leishmania* infection [13]. Prevalence studies have found infection rates of up to 25% in dogs [14], 10% in cats [15] and 50% or more in wild animals, such as rodents [16], in some geographical areas. When it comes to *Leishmania* diagnosis there are two main problems for laboratory scientists to solve. The first is that the form of the *Leishmania* parasite found in clinical samples, such as bone marrow or skin biopsies, is the 5-micron, intracellular amastigote stage. This is difficult to detect with a microscope. The other is that there is little morphological difference between many *Leishmania* species. Medical and veterinary parasitologists, along with entomologists, worked together to develop and optimise various tests. Although microscopy and serological tests are available, PCR protocols are the most useful as they provide acceptable sensitivity and specificity for diagnosis, surveillance, and research [17]. This illustrates how the One Health approach can be used to share expertise and enhance capabilities in human and veterinary medicine, as well as environmental research.

Laboratory services have a key role in surveillance for infectious diseases. Most countries routinely collect and collate data on human pathogens. Some of these infections are “notifiable,” which means that cases must be reported to a central authority by law [11]. The criteria for notification usually include laboratory confirmation of the diagnosis. Diseases are designated notifiable because, although they may be uncommon, they are likely to be serious, highly infectious and associated with outbreaks, or are included in a vaccination campaign. In the United Kingdom , the relevant agencies for notification of human diseases are the UK Health Security Agency (England) [18], Public Health Wales [19], Public Health Scotland [20], and Public Health Agency (Northern Ireland) [21]. There are also organisations analysing incidence and prevalence rates for many diseases at national and international level. These include the United States Centres for Disease Control (CDC) [22], the European Centre for Disease Surveillance and Control (ECDC) [23] and the Africa Centres for Disease Control and Prevention (Africa CDC) [24]. There are six World Health Organisation regions [25] (Africa, Americas, Eastern Mediterranean, Europe, South East Asia, Western Pacific), which collect information and coordinate with the central WHO headquarters. As well as coordinating epidemiological data, these agencies also evaluate evidence about testing methods and write policies and guidance for healthcare professionals and patients [26]. Laboratory scientists have key and varied roles in these organizations; their training, expertise and insight are invaluable.

There are analogous systems for notifying specified serious animal diseases and laboratory support is also key for these. In the United Kingdom , veterinary practitioners must report cases to the Department for Environment, Food and Rural Affairs (DEFRA) [27]. There are regional systems such as the European Union Animal Disease Information System (ADIS) [28] and the international scheme involves “listed” diseases in terrestrial and aquatic animals which must be reported to World Organisation for Animal Health [29]. It is easier to monitor infection in farm animals and domestic pets than in wild animals and birds. However, the latter are important sources of potential infection both to humans and other animals. For understandable logistical and financial reasons, projects to identify zoonotic infections in wildlife tend to focus on one type of animal (e.g., viruses in bats) or a limited geographical area (e.g., *Echinococcus* spp. in canids in Europe), but this can yield useful insights. Opportunistic surveillance of dead wildlife can also provide valuable data. For example, dead and diseased birds are sampled as part of the scheme to monitor avian influenza [30]. The global network of laboratories to test samples for infectious diseases in animals is not as comprehensive as it is for humans. Also, because it can be difficult to identify infections in non-domesticated animals, monitoring and surveillance is more limited in this group. Monitoring the health of non-domestic animals is nevertheless an important tool within One Health. The expertise of diagnostic laboratory professionals is vital for carrying out diagnostic tests, developing sampling and testing protocols and devising new methods. As the repertoire of Point of Care Tests for infectious agents expands, there is increasing interest in their use in animals and birds [31].

There is increasing recognition that, paradoxically, whilst laboratories are essential for the monitoring of health and the environment, they can be significant sources of waste. Health and safety issues as well as consideration of effective use of staff time have led to widespread employment of single use disposable equipment and pre-packaged test kit reagents. While rapid diagnostic tests offer many practical advantages, they also produce considerable volumes of plastic waste. Similarly, the disposal of masks, needles and syringes can create significant problems [32]. There is an increasing focus on enhancing healthcare professionals’ awareness of the environmental impact of their activities [33] through organisations such as the Centre for Sustainable Healthcare [34]. Diagnostic and research laboratories are starting to seriously evaluate how they use products such as plastics and potentially harmful chemicals and to find ways to reduce waste [35]. Assessment tools to facilitate this have been developed in recent years, for example, the Laboratory Efficiency Assessment Framework (LEAF) [36] in the United Kingdom and My Green Laboratory in the United States [37]. It is not always straightforward to change working practices. For example, microbiology laboratories discontinued the use of glass Petri dishes and pipettes at the end of the 20th century, because plastic was more cost effective, less wasteful of resources (since glassware required washing and autoclaving before re-use) and also safer (due to the reduced chance for sharps injuries). That considered and justifiable position taken by experienced laboratory professionals may now have to be re-visited, to reduce plastic waste. However, a recent study estimating CO_2_ emissions from microbiology laboratories concluded that the main sources were consumables and staff travelling to work [38] and made the point that laboratory grade glassware cannot be put into standard recycling systems due to its high melting point. Manufacturers and suppliers of test kits are also reviewing their practices to find ways to reduce packaging and minimise product information provided on paper. A key way for diagnostic laboratories to improve their environmental sustainability is to have the highest standards of quality management. The aim should always be to perform the most appropriate test on the correct sample, from the right patient and to report the result in good time. This idea of “diagnostic stewardship” [39] fits well with the concept of “antimicrobial stewardship.” Both are pertinent to One Health since they are approaches to using potentially finite resources carefully.

### One Health Approach to Controlling of Zoonotic Diseases

Zoonotic infections occur when humans interact directly or indirectly (e.g., via an invertebrate vector) with either wild or domestic animals that harbour mutually transmissible diseases. Control therefore requires an appreciation of both the way in which those interactions occur and the factors responsible for maintaining the infections in those animals [1–3]. The One Health approach encourages us to operate with care and humility and always be open to the possibility of unintended consequences. For example, rabies was eliminated from large parts of northern Europe, at least partly, through the dissemination of baits laced with an anti-rabies vaccine [40]. Rabies had previously been a major factor limiting fox populations and therefore their populations increased [41]. Foxes are the definitive hosts for the tapeworm *Echinococcus multilocularis* whose larval stage can infect humans within which they form potentially fatal alveolar hydatid cysts. The numbers of human infections with *E. multilocularis* have increased in several parts of Europe and one reason for this is undoubtedly the increase in the fox population [42, 43].

From a human perspective, strategies to reduce contact with potential animal reservoirs or invertebrate vectors of infection through education, along with treatment or prophylaxis programmes would be rational, but their effect has often been limited. A One Health approach, taking a wider view can be beneficial [1, 4, 7]. The malaria parasite *Plasmodium knowlesi* was first recognized in 1931 in Macaque monkeys and it was used for many years as a model for studying the pathogenesis of human *Plasmodium* spp [44]. The first case of infection in humans was recorded in 1965 in a man from the United States who had traveled to Malaysia [45]. Subsequent human cases are thought to have been under-reported for decades, while laboratory identification relied on detection of the protozoan in blood slides. This is due to the morphological similarity of *P. knowlesi* at various life cycle stages to the strictly human pathogens *P. malariae* and *P. vivax*. Once molecular methods became available in the early 2000s, it became clear that *P. knowlesi* is an important cause of human malaria in some parts of Southeast Asia [44]. In Malaysia, *P. falciparum, P. vivax* and *P. malariae* have been virtually eradicated, while reported incidence of *P. knowlesi* has risen dramatically. This rise is partly attributable to more accurate laboratory results and highlights the key role that diagnostic scientists play in patient care [44]. Since *P. knowlesi* is a zoonosis, control requires an appreciation of its transmission dynamics in wild macaques and how humans become involved in them. The vectors of *P. knowlsei* belong to the *Leucosphyrus* group of Anopheline mosquitoes, have restricted geographical ranges, and are usually found in forests [44, 46]. This means that natural transmission of *P. knowlesi* in humans is limited to rural regions of countries in Southeast Asia [47]. However, the destruction of woodland habitats has encouraged Macaques to venture into villages and towns seeking shelter and food, taking the mosquitoes with them [48]. The One Health perspective would be to understand the monkey-mosquito-human interactions. Hopefully, restoring the forests will reduce the tendency of the monkeys to enter villages and thereby reduce the incidence of *P. knowlesi* in humans [49].

The importance of taking a global view of control of zoonoses is illustrated by the outbreak of Monkeypox in 2022 [50, 51]. The disease caused by Mpox virus, which is a member of the *Poxviridae* is endemic in West and Central Africa. The virus was identified in monkeys held in a research laboratory in Copenhagen in 1958, hence the original name. The first recorded human case occurred in what was then The Congo (now Democratic Republic of the Congo, DRC) in 1970. The usual hosts for this virus are various species of rodent which live in West and Central Africa, such as the African Rope Squirrel. The infection is considered endemic in humans in countries where these rodents reside [52]. Regular outbreaks in humans occur and these have grown in frequency as have the number of people infected during the 21st century [52]. Sequencing of isolates indicate that there are two genetic clades of the virus. Clade I Mpox strains are usually associated with outbreaks in West Africa, while clade II types are mostly circulating in Central Africa [52]. Reported case fatality rates range between 2% and 10%; deaths usually occur in the very young, elderly, or immunocompromised (including HIV positive) patients. For example, between January and September 2020, there were 4,594 recorded cases of Mpox in DRC with 171 deaths, despite the public health measures in place to reduce the spread of COVID-19. This represented a 61% increase in cases compared with 2018 [53]. Investigations of the reasons for the increase in incidence of Mpox indicate that human rather than virological factors are the main causes. The Smallpox vaccine affords good protection against disease caused by its close relative Mpox [52]. Routine mass vaccination against smallpox was discontinued in the 1980s, once the virus had been eradicated [54]. This led to a gradual reduction in the number of smallpox vaccinees in the population globally, including West and Central Africa. Environmental changes have also increased population density in some places and brought more humans into closer contact with host mammals (including through handling them and eating them as bushmeat). In contrast, there is no strong evidence that alterations to the virulence of the virus contributed to changes in epidemiology since 2000 [55].

Before 2022, sporadic cases were recorded outside of Africa in people who travelled to endemic countries e.g., 3 cases of Mpox in one family in the United Kingdom after a visit to Nigeria [56] or imported animals e.g., an outbreak in prairie dogs in United States in 2003, likely to have been spread from infected rodents imported from Africa in an exotic pet shop [57]**.** MPox was rarely “newsworthy” until a global outbreak occurred which started in Europe in 2022 [50, 51]. Implementation of measures to control the disease then gained a higher priority internationally. Identifying people with the virus and isolating them requires the support of diagnostic laboratory services. The most effect laboratory investigation is PCR on samples from the characteristic lesions and other patient samples [58, 59]. It was relatively straightforward for healthcare systems in high income countries to increase the capacity for Mpox testing. The requirement for molecular diagnostics emphasizes that reported incidence in endemic countries is likely to be an underestimate. The other key tool is vaccination [55]. Vaccination of populations most at risk would be a justifiable public health measure. It would reduce both the incidence and mortality in West and Central Africa and the risk of humans acquiring the disease while visiting from other countries. A One Health approach is therefore essential for effective control of this infection through recognizing the interconnectedness of people across the world and the factors responsible for zoonotic transmission from animal reservoirs of infection [60, 61].

### One Health to Controlling Neglected Tropical Diseases

The WHO recognizes 20 neglected tropical diseases (NTDs) [62]. These are diseases caused by a range of viruses, bacteria, fungi, and parasites as well as snake bite envenomation. They are mainly transmitted among poorer communities in low and middle incomes and are considered to markedly affect women and children more than adult men. Examples of NTDs include rabies and echinococcosis, which are zoonoses where humans are infected accidently; dracunculiasis and schistosomiasis, which are caused by parasites with complicated life cycles, involving intermediate invertebrate hosts; lymphatic filariasis which is transmitted to humans via mosquito vectors and mycetoma and podoconiosis, caused by exposure to infectious agents or irritants in soil [63]. In the case of the mycobacterial infection Buruli ulcer, the organism is found in the environment but the mode of transmission to humans is still uncertain [64]. The conditions in which people are living, the quality of their food, drinking water and housing all contribute to their risk of exposure to and morbidity from NTDs [65]. Therefore, the One Health approach is important to better understanding of the epidemiology of these diseases and finding the keys to improved control. Biomedical scientists can use their expertise to play key roles in this, through laboratory diagnosis and surveillance and evaluation of test kits [66].

Chagas disease, caused by the protozoan parasite *Trypanosoma cruzi*, is endemic in many South American countries and parts of Southern United States [67]. With unwitting human assistance, it has spread to many distant places, including Australia, Canada, and parts of Europe. In endemic areas, the parasite is usually transmitted by triatomine (“kissing”) bugs belonging to the *Reduviidae* family. In other parts of the world, various reduviid bugs as well as several other blood-feeding invertebrates act as vectors [68]. Infected bugs excrete faeces contaminated with trypomastigotes of *T. cruzi* while taking a blood meal from a mammalian host. They seek shelter between rocks, in animal nests and among piles of wood or bark. They can also live in cracks in walls of poor quality housing [69]. This illustrates why an important part of One Health is to address the environment that people live in. *Trypanosoma cruzi* can also infect many species of wild and domestic mammals so, in some regions, there may be numerous reservoirs of infection [70]. Human to human transmission can occur, for example, through blood transfusion and organ donation when prior screening has not occurred [71]. This is often the case in non-endemic countries in which the risk is not recognised. Chagas disease can persist at a subclinical level for many years and therefore people who travel/migrate from endemic regions may be unaware of their infection status. In woman of childbearing age, undetected infection is a particular problem, since congenital transmission is possible [72]. Although the risk is low (around 5%), ante-natal screening is required in endemic areas.

In patients who present with suspected acute infection, laboratory diagnosis is by detection of the *T. cruzi* trypomastigotes in stained blood films or parasite DNA by PCR [73]. Most diagnoses are of chronic infection and in those cases laboratory investigation is through serology. No available serological test has sufficiently high sensitivity and specificity, so the WHO recommended gold standard method is to use two separate assays from enzyme immunoassay, haemagglutination inhibition and indirect immunofluorescence [74]. Serology or point of care tests can be used in screening (e.g., donated blood or transplant organs). It should be noted that performance of such assays is affected by the prevalence of the disease in the population [11].

There is no vaccine for Chagas Disease and the drugs used to treat it need to be taken over a long period of time and often induce unpleasant side-effects [75]. This makes patient compliance with treatment difficult and increases the chances of drug resistance developing because the parasites are exposed to sub-lethal drug titres. The non-compliance issue is exacerbated because those most at risk from primary and chronic Chagas disease are those on low incomes in badly built and poorly maintained housing [76]. These groups are least likely to access regularly healthcare interventions. The One Health approach acknowledges the effect of people’s physical environment and social welfare in terms of their potential exposure to *T. cruzi* and the development of chronic infection which can lead to long term sequelae including cardiac and GI tract pathology [77]. There is also a need to appreciate the extent to which wild animals contribute to the maintenance of Chagas disease within an area [70]. Potential for conflicts of interest can arise between conservation and disease prevention wherever wild animals are important reservoirs of infection. These can be minimised through education to emphasise the importance and value of wildlife and how to minimise potentially risky contact with infected animals.

Viral infections on the list of NTDs include dengue and chikungunya, which are both positive sense single stranded RNA viruses, spread between humans via the bite of *Aedes* spp. mosquitos, principally *A. aegypti* [78]. People infected with dengue or chikungunya can experience a range of symptoms from sub-clinical infection to a mild self-limiting illness involving pyrexia, myalgia, headache, nausea and/or rash. Some people develop a post viral-type syndrome of extreme fatigue, muscle and joint pain, debilitation and depression which can last for months or years. The most serious possible sequelae are neurological and can be fatal. Reported case fatality rates for these infections vary but can be around 10%. There are 4 serotypes of dengue virus and Dengue haemorrhagic fever (DHF) occurs when a person who has recovered from a previous infection with one serotype is infected with a different one; this can lead to an overwhelming inappropriate immune response [79]. The reported incidence of both these diseases have been increasing during the 21st Century. This is partly explained by increased surveillance and better diagnosis. Laboratory testing for the viruses has made an important contribution to this since it is hard to distinguish infections with chikungunya, dengue and zika viruses clinically. PCR, ideally multiplex assays are the optimal diagnostic approach [80]. However, these are expensive and not widely available in endemic areas. Point of care tests for dengue IgM have proved useful, particularly during outbreaks, but their sensitivity can be variable and for some kits unacceptably low (reviews suggest between 13.8% and 90%) [81]. Also of note is the limitation that IgM titre does not reach detectable levels until a week or so after the onset of symptoms. Vaccines to protect against DHF are becoming widely available, but they are only applicable for people who have previously recovered from a primary infection. Since most dengue infections are sub-clinical, the WHO recommends IgG screening of potential vaccinees. With reported sensitivities of over 95%, point of care tests can be helpful [82]. For diagnosis of acute primary infection POCT tests designed to detect the non-structural protein 1 (NS1) viral antigen are reported to have greater sensitivity [83]; performance appears to be improved further for kits combining NS1 with IgM detection. In contrast, while point of care tests for chikungunya infection are under development and evaluation, they are not yet widely available [84]. This is likely to mean that incidence is under reported.

Humans are the main reservoir hosts for both dengue and chikungunya (although surveys have detected evidence of infection in some wild animals) so the One Health approach to control is useful as it takes the environmental factors into account [85, 86]. Climate change is likely to be causing an increase in people’s exposure to vector mosquitoes. Analysis of available data suggests that prolonged higher global temperatures are associated with a rise in reported incidence of dengue [87]. An effect on mosquito behaviour has been noted in that the heat seems to induce them to reproduce more often, so need to take more blood meals. Another important issue is flooding; *Aedes* spp. mosquitoes prefer to lay their eggs in water containing organic matter (i.e., dirty water) and pools of water which form from receding flood water are ideal places for larvae to develop [88]. Therefore, control of dengue and chikungunya infections should include public health measures to attend to the environment in endemic areas [89]. There should be regular maintenance of water courses, to remove possible mosquito breeding sites, along with effective clean up after floods. Better prediction of extreme weather events could be helpful. This would allow plans to be put in place to move people to safety in time and to provide them with good shelter and food in their temporary living conditions. Then, they should be less likely to return to their homes before suitable maintenance of the environment has been carried out.

Trachoma results when the eyes are infected with the bacterium *Chlamydia trachomatis*. The condition was once widespread throughout the world but is now mostly restricted to poorer rural communities in parts of sub-Saharan Africa although there are also problems in parts of the Middle East, Australasia, and South/Central America. Nevertheless, *C. trachomatis* remains the most significant infectious disease that causes vision loss and blindness [90]. Repeated infections of the inner eyelid over time causes scarring that results in the eyelashes being pulled inwards so that they scrape over the corneal surface thereby causing further inflammation and scarring. Trachoma is intensely painful and this, coupled with the compromising of vision, reduces a person’s ability to study or work and thereby contributes to poverty. The bacteria are typically spread passively by flies, such as *Musca sorbens*, that are naturally attracted to feed around the eyes’ secretions and nasal discharges although they can also be spread via clothing, bedding, or on the hands. The disease is therefore particularly common in communities that lack access to safe water supply in which people can wash themselves and their clothes. Incidence is also high in women, because they are usually responsible for handling clothing and bedding, and young children. Tackling the problem of trachoma requires a combination of approaches [91]. Firstly, treating those who are already infected to relieve their suffering and reduce the chances of them being a source of infection to others. This can be done by a combination of antibiotic treatments and, in extreme cases surgery to treat the disabling scarring (“trachomatous trichiasis”). The One Health approach acknowledges the importance of the living conditions in control of this infection. People should have access to safe, uncontaminated water sources for drinking and washing. Improved sanitation would reduce the opportunities for the flies to breed. Education to explain the importance of face-washing and sanitation are also necessary. This forms the basis of WHO’s “SAFE strategy” for combating trachoma. The acronym stands for: Surgery for people with extreme scarring, Antibiotic treatment for active *C. trachomatis* eye infections, good facial hygiene, and improvement of the environment. The aim is to eliminate trachoma, which therefore requires a long-term engagement with those at the most risk from the disease, considering the people, insects and environmental factors [92, 93].

### One Health Approach to Combatting Antimicrobial Resistance

Antimicrobial resistance is one of the most serious threats that will impact us in the coming years. Many pathogenic bacteria, e.g., *Mycobacterium tuberculosis* and *Staphylococcus aureus*, are becoming increasingly resistant to all our currently available antibiotics [94]. This will impact on everything from routine operations to the re-emergence of diseases in countries from which they had been largely eliminated [95]. Although the development of resistance is a natural phenomenon and to be expected to occur over time, the speed of development and spread of resistance among numerous pathogens is largely a consequence of the ways antimicrobial and antiparasitic drugs have been, and continue to be, misused. Indeed, in his Nobel Prize acceptance speech, Alexander Fleming, presciently predicted that bacteria would develop resistance to penicillin if the drug was not used more wisely [96]. He was soon proved right. Furthermore, unless a patient is in hospital, they are assumed to consume the required doses at the appropriate intervals. This is often not the case which results in microbes being exposed to sub-lethal levels and this contributes to the selection of resistance genes. This is not just an issue for antibiotics and there is a suggestion that Merck’s COVID antiviral molnupiravir (brand name Lagevrio) can result in virus mutations that subsequently spread to other people—although there is no evidence to date that these mutations affect pathogenicity or transmissibility [97].

Everyone knows that antibiotics have been oversubscribed for many years. In the United Kingdom , it is now supposedly necessary to obtain a prescription from a medical doctor or other qualified practitioner to obtain an antibiotic. Although doctors are becoming less willing to prescribe antibiotics, they are often subjected to pressure from patients to prescribe them where they are not necessary. Unfortunately, even when antibiotics are needed, tests are not always undertaken to determine whether the antibiotic prescribed will be effective. It would be helpful if more accurate point of care tests that could determine species/subtype and also drug sensitivity could be developed. A further problem in the United Kingdom is that it is possible to obtain antibiotics online by simply filling in a questionnaire. In some countries there is an even laxer approach. There is therefore a need to either change legislation or enforce existing legislation more stringently both in the United Kingdom and elsewhere in the world.

The combination of over-prescription and incorrect consumption of antimicrobials results in massive amounts of them, and their metabolic products, being excreted and entering the sewage system. Additionally, many prescribed antibiotics that remain unused end up being flushed down the toilet or washed down the sink. Within the sewage system the antimicrobials interact with numerous microbes and become dispersed into the wider ecosystem; even in the United Kingdom there are regular releases of raw sewage into waterways and the sea. Interestingly, the presence of microplastic pollutants in the soil has been linked to the spread of antimicrobial resistance [98]. This may be through bacteria possessing genes conferring antimicrobial resistance forming biofilms around the microplastics and thereby avoiding being washed through the soil; the microplastics then become associated with crops and/or ingested by farm animals [99]. Another possibility by which antimicrobial resistant bacteria can enter the food chain is by becoming attached to even smaller plastic particles, nanoparticles, that are absorbed by plant roots and then transported around the plant [100]. Microplastics are now ubiquitous within aquatic and terrestrial ecosystems and, consequently, the food and water we consume, and their direct and indirect effects our health and that of other living organisms are not yet understood [101].

The widespread use of antibiotics means that many non-pathogenic microbes become exposed to them, both within our bodies, such as in the gut microbiome, and in the wider environment. Consequently, numerous harmless microbes also develop antibiotic resistance genes, and these can then find their way into pathogenic microbes through horizontal gene transfer [11]. For example, drug resistance genes steadily accumulate within our gut microbiome over our lifespan, and within microbes associated with the food we consume. Therefore, there is a possibility that these might be transferred to pathogenic species whilst we are undergoing antimicrobial therapy, thereby conferring resistance to them. It is therefore important to limit our own exposure to antimicrobials and their entry into the environment [9]. It is also a good idea for those who are on antimicrobial therapy to be careful about what they eat and how it is prepared to reduce the chances of consuming microbes carrying antimicrobial resistance genes.

The misuse of antibiotics within human medicine has been compounded by massive misuse of the same (or closely related) antibiotics within farming. Indeed, agriculture accounts for most of the consumption of antibiotics in many countries, sometimes as much as 80% [102, 103]. Some estimates suggest that the combined medical and veterinary usage is responsible for 100,000–200,000 tonnes of antibiotics entering the global environment every year [104]. Many of the microbes that are pathogenic in domestic animals are also pathogenic in humans. Therefore, there is potential for resistant strains to develop in domestic animals and subsequently infect humans [105].

In many cases, agricultural use of antimicrobials has not been associated with the treatment of diseases but as a means of increasing productivity. For example, “dry cow therapy” aims to prevent mastitis and increase milk yield at subsequent lactation [106]. It involves giving cows a constant dose of antibiotics regardless of whether they are suffering from any infections. This results in large amounts of antibiotics entering the environment; up to 80% of an antibiotic can be excreted in the urine and faeces. The use of antibiotics as growth promotors was banned in the European Union in 2006 but the practice remains common in many countries, particularly in the rearing of poultry [107]. There is also considerable use of antibiotics for disease prevention and as growth promotors within aquaculture, which leads to contamination of the ecosystem and fish/shellfish consumed by humans [108]. Although there are increasing calls to ban the use of antibiotics as growth promotors in the United States [109], at the time of writing, their use was still allowed. This has major implications for the trade of agricultural produce worth millions of pounds per annum between countries and emphasises the importance of both national and international politics in determining legislation concerning the use of antimicrobials. The lack of consensus between countries concerning the agricultural use of antimicrobials is unfortunate since enforced banning of antibiotics as growth promotors can have rapid beneficial effects. For example, in China the banning of colistin as a growth promotor quickly led to a decline in mcr-1 (colistin-resistant) *Escherichia coli* in pigs in 14 provinces from 45% to 19% within 2 years and declines were also found in humans and environmental samples [110].

Drugs used in veterinary medicine are also often misappropriated to treat humans. When rumours spread that the antiparasitic drugs levamisole and ivermectin (widely used in veterinary medicine) were effective in the treatment of COVID these drugs rapidly sold out in many countries. Indeed, in South America, it became impossible to conduct double blind studies to determine ivermectin’s effectiveness because so many people had already taken the drug [111]. Whether this will have consequences for the development of resistance among helminth parasites is not yet known. Similarly, various antimalarial drugs were promoted, with very little evidence, as being effective against COVID and this led to many people taking these [112]. In countries in which malaria is endemic this could further the development of resistance among *Plasmodium* species.

Although reducing the prescription of antibiotics (and antiparasitic drugs) by medical and veterinary professionals is obviously important, this is negated if they continue to be made widely available in pharmacies and other outlets. In addition, as with virtually everything, if there is a demand then there is always someone willing to supply it on the internet. There is also a problem in many developing countries with the sale of contraband antibiotics and antiparasitic drugs. These often contain lower levels of the active ingredients or are incorrectly formulated. These are believed to have contributed to the rapid development of resistance of *Plasmodium falciparum* against artemisinin in parts of Asia [113].

Despite the acknowledged role that agriculture plays in the spread of antimicrobial resistance, its consumption is predicted to increase considerably in forthcoming years [114, 115]. It is therefore going to take a One Health approach involving multidisciplinary approaches and cooperation of numerous national and international agencies to combat the problem.

### One Health Approach to Ensuring Food Safety

Food security and food safety are important for both humans and animals. The presence of toxins, pollutants or pathogens in material destined for consumption is not always immediately apparent. This highlights the importance of mechanisms for regular testing of foodstuffs to ensure that they meet set standards and to ensure appropriate storage and transport. Where a specific risk has not been identified, it may be overlooked in quality measures. The One Health approach recognises the effect of interactions with animals and environmental conditions on whether particular items present a risk to humans when eaten. For example, as outlined in *One Health to Controlling Neglected Tropical Diseases* section, Chagas disease is normally transmitted by blood-feeding reduviid bugs. However, in parts of South America, there have been outbreaks of the infection caused by the consumption of fruit juice [116]. Investigations indicated that the drinks had been prepared from fruit contaminated with faeces from bugs infected with *T. cruzi*. The reduviid bugs had acquired the parasite from wild animals and subsequently defecated on the fruit. Fruit juices prepared for export are sterilized and would therefore pass any safety checks. However, fruit juices prepared for local consumption are often not heat treated or subject to quality tests, meaning that the risk had not been noticed until people became ill [117].

Laws and guidance on food storage and preparation may have little impact if there is not the means to undertake them or they present conflicts with traditional practices. Across the world there are many people who are unable to afford a refrigerator, or where the electricity supply is cut off for prolonged periods. Consequently, there is an increased risk of bacterial food poisoning. Similarly, food cannot be cooked if there is not the means to cook it. Tradition also plays an important part in how food is prepared. For example, consumption of raw or undercooked meat is common in many countries but can lead to infection with parasites such as *Trichinella* spp. and *Taenia spp.* as well as various bacterial pathogens [118]. The risk is greater if the meat did not come from animals subject to meat inspection and/or the meat was not stored appropriately [119]. From a One Health point of view, the risks of hunting and then eating wild animals should be noted. For example, people are often unaware that wild boars in Europe can be a source of *Trichinella* spp. A recent outbreak of *Trichinella britovi*, which affected at least 35 people in Northern Italy, was linked to consumption of raw sausages prepared from the meat of a single animal [120].

Outbreaks of food poisoning can arise from infected catering workers who prepare the food. Sometimes, they are not aware that they are infected, or they are suffering a mild infection that they dismiss as unimportant [121]. However, most food poisoning outbreaks result from incorrect handling, cooking, or storage of food [122]. There is therefore considerable interest in developing point of care lateral flow tests that could be used in the food industry to detect the most common causes of food poisoning [123].

All organisms have associated microbiomes, and their compositions have implications for health [124]. Humans have distinct microbiomes associated with different regions of our body and these perform various functions including contributing to the innate immune response. Perturbations in gut microbiome (dysbiosis) have been linked with conditions ranging from diarrhoea to mental illness [124, 125]. Frequently, dysbiosis manifests as a reduction in the diversity of gut microbiota [126]. This is often ascribed to what is often called Westernized lifestyles but could be more accurately be called a consequence of living in an urban environment with access to good sanitation and medical care and consuming a diet that is high in processed foods [127]. This is an almost inevitable lifestyle for many people living in countries with developed economies. Some authors have even suggested that urbanization “could be a public health threat” [128] through its impacts on our ability to acquire and maintain the diverse microbial communities considered necessary for many aspects of health. Nevertheless, most humans now live in urban areas from towns up to megacities and there is no prospect of this changing. Research suggests that geographical location and level of urbanisation can account for variation in people’s gut microbiome [129]. Therefore, the One Health approach to evaluation of environmental factors might help to identify appropriate means of counterbalancing the downsides [130]. Nevertheless, a cautionary note is needed because there is an ongoing debate over the methodology used to analyse and interpret microbiome data that impacts on the results obtained [131]. There is therefore a concern that some of the claimed linkages between microbiomes and health may have been overstated [132].

The bacterial phyla which tend to dominate the composition of the mammalian gut microbiome (Firmicutes, Bacteroidetes, Proteobacteria, Actinobacteria) are also associated with roots of plants [133]. Consequently, the plant microbiome could potentially influence the composition of an animal’s (or human’s) microbiome directly through herbivory or indirectly through consuming herbivores. However, more research is needed [134]. Many animals, especially herbivores, also consume appreciable amounts of soil unintentionally whilst grazing or intentionally through geophagy. The plant microbiome is itself impacted upon by the wider soil microbiome and both can be altered significantly by herbicides and fungicides and there are increasing concerns over antibiotics used in veterinary and human medicine finding their way into the wider ecosystem. This opens the possibility of facilitating the transfer of genes coding for antibiotic resistance and the proliferation and transfer of pathogenic bacteria and fungi [135]. One should not, however, consider microbial transfer solely in a negative context because it is a normal ecosystem process—problems are most likely to occur because of unintended human actions.

Cryptosporidiosis is a parasitic infection, found in numerous wild and domestic mammals, birds, reptiles, amphibia, and fish, as well as humans [136]. It results from infection with protozoa belonging to the genus *Cryptosporidium* of which there are numerous species. Most human infections are associated with infection with either *Cryptosporidium hominis*, for which humans are the usual host, or *Cryptosporidium parvum* whose normal hosts are mice. Even within a single species there are numerous sub-types that vary in their distribution, pathogenicity, and host range. In adult humans, cryptosporidiosis usually manifests in self-limiting diarrhoea and intestinal cramping and is seldom serious but in children less than 5 years-old or immunosuppressed patients, it is potentially fatal. Infections are normally acquired through ingesting the oocyst stage through faecal-oral contamination. Outbreaks are therefore commonly associated with contamination of local water supplies or swimming in contaminated lakes and recreational pools [137]. Because *Cryptosporidium* species infect numerous wild and domestic animals and many are zoonotic, it is difficult to prevent water sources from becoming contaminated. Effective water treatment is therefore essential. The risks of transmission are likely to increase where climate change causes more frequent and/or heavy rainfall [138]. For example, even within a developed country such as the United Kingdom , downpours often lead to the discharge of untreated sewage into water courses and the sea—and the oocysts can survive in seawater [136].

Because cryptosporidiosis causes non-specific symptoms it is important to distinguish it from other causes of gastrointestinal tract infections. Although the oocysts can be identified in stools, they are extremely small (5–7 µm) and difficult to detect even with the aid of the modified Ziehl-Nielson stain technique. In addition, the oocysts are shed sporadically so several stool samples collected over a period of days need to be examined. Detection of the parasite DNA through PCR testing is more sensitive and specific. It is important to determine species and subtypes when investigating outbreaks to understand the source and implement suitable control measures [139]. The One Health perspective is helpful [140]. For example, where the source of the infection in humans is water contaminated by run off from farmland, the environment in which the animals are being kept is also a contributory factor.

Bushmeat is meat derived from wild terrestrial animals that are not traditionally considered game animals [141]. Game animals are those hunted for sport as well as for food (e.g., grouse, red deer, elk). Bushmeat therefore comes from numerous animals including iguanas, pangolins, fruit bats and gorillas. Some people lack alternative sources of protein and hunting is their traditional way of life, whilst for others bushmeat is a preferred food. As people move from rural to urban areas or migrate to other countries, they often retain a desire to continue consuming bushmeat. This may be a consequence of taste, tradition, or linked to religious practices. It may also be seen as a status symbol, with increased affluence enhancing the ability to obtain certain meats. Consequently, those involved in the trade can cater for both national and international markets; this is aided by rapid road and air transport, mobile phones, and the internet. Much of the trade in bushmeat involves protected species and therefore the killing is done covertly and illegally. Consequently, it is difficult to obtain accurate figures for amount of bushmeat being harvested but it is undoubtedly enormous [142]. Most of the bushmeat trade is domestic and only a small proportion is exported. Nevertheless, significant quantities, most of it undetected, enter Europe every year through trafficking by both individuals and large criminal organisations. For example, a freedom of information request made by the Daily Mirror to the Home Office revealed that 1,149 kg of bushmeat was seized by Border Agency staff at United Kingdom airports in 2018/19 and this represented a considerable increase on previous years [143]. Indeed, at Heathrow in 2019, they recovered over a ton of bushmeat on a single plane flying from West Africa and on its way to the United States [144].

Zoonotic infections potentially acquired from bushmeat include metazoan parasites, such as the nematode *Trichinella spiralis*, bacteria, such as *Brucella* spp., *Campylobacter* spp., and *Vibrio* spp., and viruses such as Ebola and Marburg virus [145]. In 2012, Smith *et al* [146] provided the first demonstration that non-human primate bushmeat illegally imported into the United States tested positive for simian foamy viruses and/or herpes virus (cytomegalovirus and lymphocryptovirus). The importance of simian foamy virus as a human pathogen is uncertain but it is transmissible between non-human primates to humans. Simian foamy virus is a retrovirus and there is concern that it may have a similar potential to simian immunodeficiency virus (SIV) to cross the species barrier; SIV is thought to have evolved to become HIV. Although they did not recover SIV, many of the primates in Cameroon are infected with various SIV phylogenetic lineages [147]. Similar infection levels can be expected in primates in other African countries, and they therefore present a potential infection risk to those who kill, handle, and consume them [148, 149].

Several outbreaks of Ebola virus in Gabon (West Africa) have been linked to the butchering and consumption of chimpanzees that were found dead [150, 151]. Bats are commonly consumed as bushmeat and represent another source of infection [152]. Similarly, severe acute respiratory syndrome virus (SARS) was first identified in 2003 when it was responsible for a worldwide epidemic that caused about 8,000 cases and 750 deaths [153]. The virus is thought to have originated from masked palm civets (*Paguma larvata*) that are sold for food in the markets of southwest China [154]. The disease is often spread through infected droplets that are coughed or sneezed out. After being transmitted from palm civets to humans the disease then spread rapidly between humans, aided and abetted by air travel. The disease was brought under control, but any new cases have the potential for starting another epidemic. Although masked palm civets are often stated as the source of the 2003 SARS epidemic, they were almost certainly not the natural reservoir of the virus. Many of the civets traded in Chinese markets are not caught in the wild but are commercially farmed. Therefore, it is debateable whether they are really “bushmeat.” In addition, very few of both the farmed and wild civets have tested positive for the disease. Instead, the virus reservoir is thought to be bats, which are known to harbour a much higher diversity of coronaviruses than other mammals [155].

Although acquiring transmissible diseases from wild animals through the consumption of bushmeat is a serious concern, it is far more likely that people would contract food poisoning. This is because the animals are often killed in hot tropical forests and therefore start to decompose rapidly, and they may not be eviscerated for around 24 h [156]. Even if the meat is smoked or dried it is seldom kept in hygienic conditions.

Most countries have laws prohibiting the killing and exploitation of wildlife, but these have only a limited impact on the bushmeat trade. Similarly, there is legislation concerning the importation of meat from domestic animals, but logistical and resourcing issues severely limit the extent to which these are enforced. Both trades pose serious health risks to both humans and livestock and therefore a One Health approach is beneficial, starting with a need to appreciate the socioeconomic factors that underly them. For example, a study in Sub-Saharan Africa found that young males and mature females were particularly at risk of contracting Simian T-lymphotropic virus (STLV-1) [157]. This is likely to be because younger men are involved in hunting monkeys and older women in preparing meat from them. It is important to understand the nutritional, cultural and environmental influences on people’s decisions to choose bushmeat. There are consequences for the animal populations—not only through being preyed on, but also potential transmission of infections from humans to animals [158]. People who consume bushmeat and illegally imported meat need to understand the risks it poses to their health. The availability of rapid techniques to identify the type of meat as well as its microbiological profile would be useful because illegally imported meat/bushmeat and its provenance are often mislabelled. Similarly, diagnostic laboratory scientists need to be aware of the potential for associated diseases caused non-endemic pathogens.

### One Health Approach for Ensuring Environmental Health

Climate change is an increasingly significant factor in public health [159]. It affects all communities, and its consequences will undoubtedly become more serious in the coming decades. How it manifests varies between locations but can be broadly categorised as a prolonged rise in air and sea temperatures accompanied by extreme weather events, such as heatwaves, floods, cyclones, and droughts [160]. These, in turn, destroy infrastructure, render people homeless, and reduce the production of crops and livestock. Similarly, rises in sea temperatures and acidification, in combination with rising sea levels is devastating fish stocks and causing land erosion and flooding in coastal regions. These problems are increasingly apparent in higher income countries, but the consequences are far more serious for poor communities in low- and middle-income countries where they are leading to poverty, malnutrition, and economic migration [161, 162].

Climate change can influence transmission of infectious diseases in numerous ways and the One Health approach is useful to understanding how factors are connected [163, 164]. Increases in temperature on land affect humans and other vertebrates by making some environments inhospitable to the point of being uninhabitable. This induces migration, potentially bringing people and animals into closer contact than previously and this is an opportunity to spread zoonosis and indeed anthroponoses. The extreme heat will have an overall detrimental effect on the health of those unable to relocate, not only because the body is attempting to survive in a higher temperature but also because of food scarcity due to crop failure. It is possible that this could weaken the immune response, making individuals more vulnerable to infections such as urinary tract infection and septicaemia [165]. Another consequence of heat waves is people and animals seeking to cool down in water. This can bring them into contact with water borne pathogens such as *Leptospira* spp. [166] and *Acanthamoeba* spp. [167] and also, potentially, increase contamination. Reported incidence of both these infectious diseases has been rise around the world during the 21st century. Some of this increase is due to improved laboratory diagnosis, aided by the refinement and greater availability of molecular based assays. However, the One Health approach would also consider the environmental changes involved [168]. For invertebrates, higher prevailing temperatures may affect their life cycles. For example, provided there is sufficient moisture, hookworm larvae can withstand land temperatures of up to 40°C and their developmental cycle can take place three times more quickly than usual [169]. This would enhance the opportunities for more human infections. Mosquitoes could also go through their life cycles faster, increase their geographical range and potentially overwinter in places currently considered to be too cold, as global temperatures increase [170].

The other big effect of climate change, which is possibly of greater significance for infectious disease control, is the increased frequency and extent of very heavy rainfall leading to flooding. For humans and vertebrates, the consequences are similar to those outlined for extreme heat—movement of populations, destruction of shelter and food crops. Rather than seeking contact with contaminated water to cool down, people and animals will be surrounded by river water mixed with sewage, which brings the risk of diseases such as cholera [171]. Immediately following a flood, the only available drinking water might be sources in which faecal-oral pathogens are concentrated. Receding water also leaves pools in new places which are attractive sites for mosquitoes to lay eggs. Coupled with the effect of global warming this can increase the spread of a range of mosquito borne infections [172].

The example of malaria illustrates the uncertainty in predicting the effect of climate change and how the One Health approach, particularly taking environmental factors into account is important. The life cycle of the parasites which cause malaria, *Plasmodium* spp. is complex and involves certain *Anopheles* spp. mosquitoes as the definitive host (site of sexual reproduction) with humans or other vertebrates, depending on the parasite species, as the secondary host. Changes in the environment may affect the vertebrate and invertebrate hosts differently, so assessing whether malaria will become a more serious problem in endemic countries and/or spread to new geographical regions where it is currently absent or not a serious problem is complicated [173]. Obviously, increases in temperature and rainfall can be expected to make the environment more suitable for the for the mosquito vectors of malaria. The same would be true for mosquito species that spread other diseases, such as dengue fever and certain filarial nematodes. Whilst some computer programmes that model the impact of climate change on malaria transmission support this prediction, there are many other factors in addition to mosquito abundance that influence the spread of the disease [174]. Furthermore, global climatic changes are not acting in isolation. For example, local factors, such as deforestation can change the suitability of a region for certain mosquito species and may itself cause local climatic changes. Societal factors are also important for disease transmission. For example, although malaria transmission no longer occurs in the United Kingdom and Northern Europe, up until the early 1900s it was a common disease [175]. The decline in transmission was unrelated to changes in the climate or the disappearance of anopheline mosquito vectors. Therefore, theoretically, malaria could re-establish itself. Indeed, thousands of people who are infected with malaria arrive in the United Kingdom every year, but forward transmission does not occur. This is, in part, because of improvements in the standard of living and drainage of marshland. In addition, those expressing symptomatic malaria are quickly identified and treated so they rarely encounter a species of mosquito capable of transmitting it.

Taking the One Health perspective to understand pathogens can be useful in the context of alterations to the environment brought about by climate change. Schistosomiasis is caused by the helminth parasites in the *Schistosoma* spp. For the species which are human parasites, sexual reproduction takes place in humans and asexual reproduction occurs in very particular, geographically restricted species of aquatic snail [176]. There is some evidence that incidence of schistosomiasis could be decreased in certain areas by climate change due to the flooding displacing the snails (although in others there could be increases) [164]. So, while epidemiologists and laboratory diagnostic microbiologists should be prepared to monitor some infections as they spread more widely around the world, there may be some surprising reductions in others.

## Conclusion

The examples discussed here highlight the value of the One Health approach to investigating the causes, epidemiology, and control of infectious diseases. Biomedical Scientists and Clinical Scientists working in diagnostic laboratories understand the value of working in multidisciplinary healthcare teams to review complex clinical cases. There is a clear case for an analogous approach within One Health. To address the complex, multifactorial issues of infectious diseases requires inputs from a range of healthcare professionals, veterinary practitioners, social workers, and ecologists. The stated aims of the One Health Quadripartite initiative include promoting a cross sectorial approach and producing advice and policy guidelines for governments, healthcare planners and relevant national and international organisations. This must include funding to support the design of multidisciplinary research programmes and robust evaluation of their outcomes. Schemes need to focus on particular challenges in single geographical areas or specific infections, in order to produce reliable evidence. However, they should also be global in scope, since the problems associated with infectious diseases are steadily increasing across the world. The optimal consequence of taking a One Health approach would be to encourage everyone to “think global, act local.”
